# Point-of-care uric acid testing is useful in routine clinical care of gout

**DOI:** 10.1186/s13075-019-1891-1

**Published:** 2019-05-09

**Authors:** Philip L. Riches, Kristen Sing, Kathryn Berg

**Affiliations:** Institute of Genetics and Molecular Medicine, University of Edinburgh, Edinburgh, EH4 2XU UK

**Keywords:** Gout, Point-of-care, Uric acid meter

Fabre et al. recently reported that the HumaSens^plus^ point-of-care uric acid (UA) meter performed well in comparison to venous UA in a cohort of 238 diabetic patient [1]. We have performed a service evaluation of the HumaSens2.0^plus^ UA meter in patients seen in a specialist gout clinic for investigation or review. All patients attending the clinic from August 2017 onward were offered fingerprick testing with results compared to a venous sample analysed using an Abbott colorimetric uricase assay as part of standard care. Summary characteristics of the patients included in the study are given in Table 1 with complete data available in Additional file 1.

**Table 1:** Characteristics of study population (*n* = 131)

Characteristic	Value
Gout	124 (94.6%)
Visible tophi	41 (31.3%)
Age (years)	58.1 (± 16.2)
Male sex	110 (84.0%)
Weight (kg)	91.9 (± 21.7)
Alcohol (u/week)	9.7 (± 13.2)
Diuretics	25 (19.1%)
Anti-hypertensive medication	44 (33.6%)
Urate lowering therapy	98 (74.8%)
Renal impairment	eGFR < 30 ml/min/1.73m^2^ 2 (9.1%) eGFR 30–60 ml/min/1.73m^2^ 23 (17.5%)

Similar to the results of Fabre et al., we observed a close correlation between the capillary and venous UA levels (Fig. 1). We observed discrepancies which would influence treatment escalation decisions in just five individuals who had levels of UA close to the 0.3 mmol/l threshold and seven individuals around the 0.36 mmol/l threshold. The performance of capillary uric acid measurements in identifying hyperuricaemic individuals was analysed using ROC curve analysis with an AUC of 99% for detection of venous UA levels above 0.3 mmol/l (Fig. 2a) and an AUC of 98.5% for detection of venous UA levels above 0.36 mmol/l (Fig. 2b). These results compare favourably with those reported by Fabre et al. and possibly reflect improved performance of the HumaSens2.0^plus^ device over the earlier HumaSens^plus^ device. We also evaluated the role of haematocrit as this was the only potential confounder identified by Fabre et al. Although haematocrit was associated with capillary UA levels independently of venous UA levels in our population, the addition of haematocrit to a prediction model of venous UA levels yielded a trivial increase in performance (ROC curve analysis AUC 99.2% for UA levels greater than 0.3 mmol/l, and unchanged AUC 98.5% at the threshold of 0.36 mmol/l). On four occasions, ‘Lo’ error reading was obtained prompting immediate retesting. Three continued to give a ‘Lo’ reading with all these confirmed on venous testing as being below the reference range of the meter (0.18 mmol/l). The remaining ‘Lo’ error reading corrected on repeat testing.

**Fig. 1:**
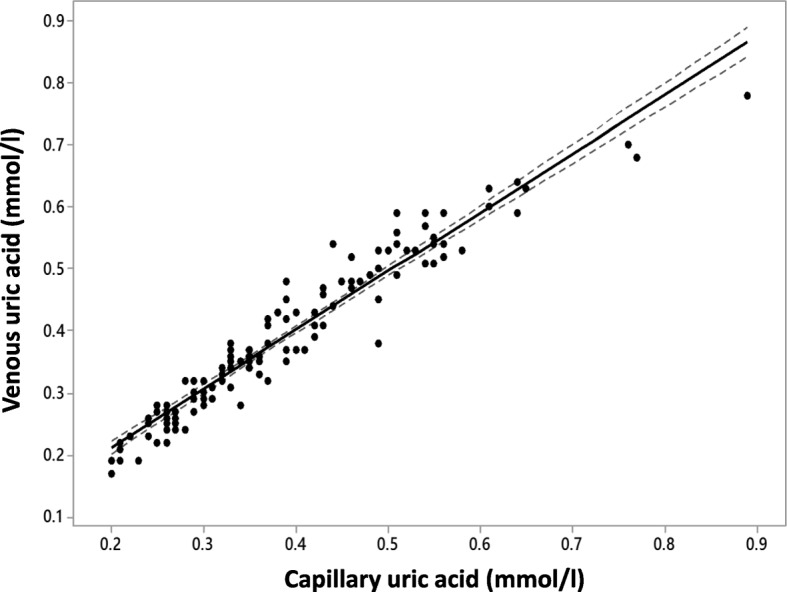
Correlation between capillary and venous uric acid measures. Regression line with 95% confidence intervals is shown. Pearson correlation coefficient 96.4% (*p* < 0.001)

**Fig. 2:**
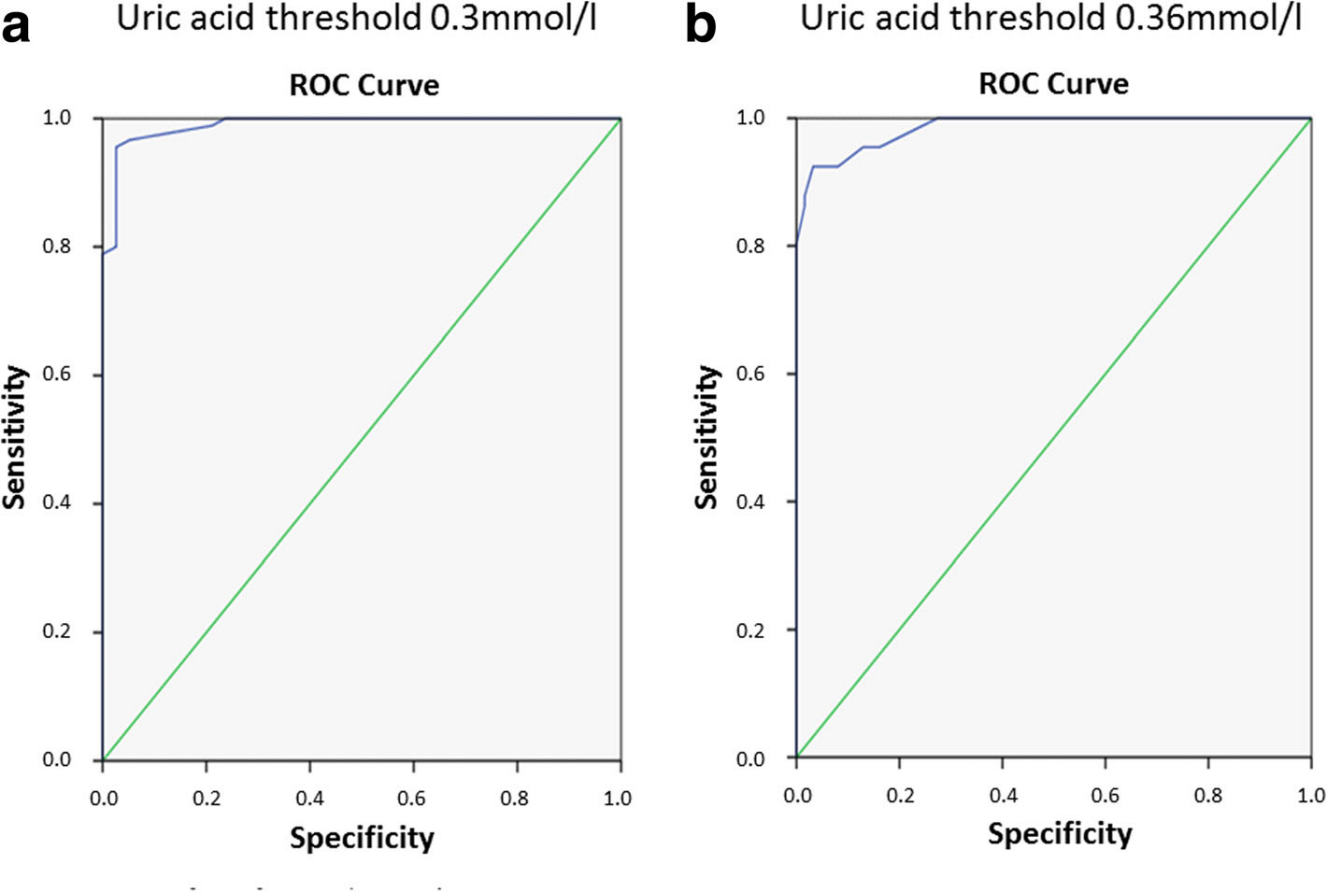
ROC curve analysis of fingerprick uric acid (UA) measurements in identifying individuals with hyperuricaemia defined as venous UA greater than 0.3 mmol/l (**a**, AUC 99%) or greater than 0.36 mmol/l (**b**, AUC 98.5%). Use of a fingerprick threshold of 0.3 mmol/l results in 97.8% sensitivity and 86.8% specificity for venous readings above this same level (within this cohort 100% sensitivity was given by a capillary threshold of 0.28 mmol/l and 100% specificity by a capillary threshold of 0.35 mmol/l). Similarly, fingerprick readings at the 0.36 mmol/l threshold yielded 92.4% sensitivity and 91.9% specificity (with 100% sensitivity seen at a threshold of 0.33 mmol/l and 100% specificity at 0.4 mmol/l)

Our results confirm the findings of Fabre et al. and confirm the reliability of the HumaSens2.0^plus^ point-of-care device in a population of gout patients. For the vast majority of patients, these results can reliably inform discussions around treatment compliance and the need for additional therapy, as well as allowing direct prescriptions of additional treatment without the need to recall patients.

## Additional file

Additional file 1: Raw data from gout clinic cohort. (XLSX 27 kb)

## Abbreviation

UA: Uric acid
